# Like No Other? A Family-Specific Network Approach to Parenting Adolescents

**DOI:** 10.1007/s10964-023-01912-5

**Published:** 2023-12-06

**Authors:** Savannah Boele, Anne Bülow, Adriene M. Beltz, Amaranta de Haan, Jaap J. A. Denissen, Marleen H. M. de Moor, Loes Keijsers

**Affiliations:** 1 Department of Psychology, Education and Child Studies, Erasmus University Rotterdam, Rotterdam, the Netherlands; 2 Department of Psychology, University of Michigan, Ann Arbor, MI USA; 3 Department of Developmental Psychology, Utrecht University, Utrecht, the Netherlands

**Keywords:** Parenting, Adolescence, Intensive longitudinal data, Heterogeneity, Idiographic, Person-specific

## Abstract

Numerous theories suggest that parents and adolescents influence each other in diverse ways; however, whether these influences differ between subgroups or are unique to each family remains uncertain. Therefore, this study explored whether data-driven subgroups of families emerged that exhibited a similar daily interplay between parenting and adolescent affective well-being. To do so, Subgrouping Group Iterative Multiple Model Estimation (S-GIMME) was used to estimate family-specific dynamic network models, containing same- and next-day associations among five parenting practices (i.e., warmth, autonomy support, psychological control, strictness, monitoring) and adolescent positive and negative affect. These family-specific networks were estimated for 129 adolescents (*M*_age_ = 13.3, *SD*_age_ = 1.2, 64% female, 87% Dutch), who reported each day on parenting and their affect for 100 consecutive days. The findings of S-GIMME did not identify data-driven subgroups sharing similar parenting-affect associations. Instead, each family displayed a unique pattern of temporal associations between the different practices and adolescent affect. Thus, the ways in which parenting practices were related to adolescents’ affect in everyday life were family specific.

## Introduction

Parenting adolescents involves a dynamic interplay between a variety of parenting practices and adolescents’ well-being (Bronfenbrenner, 2005; Darling & Steinberg, 1993). While it is widely theorized that parent-adolescent dynamics vary across families (e.g., Belsky & Pluess, 2009; Smith & Thelen, 2003), there are divergent ideas about how and why these dynamics might vary. On one hand, it has been suggested that the nature of parent-adolescent dynamics varies from subgroup to subgroup; for instance, due to the child’s personality (Pluess, 2015), legitimacy beliefs of parental authority (Darling et al., 2007), a parent’s stable parenting style (Darling & Steinberg, 1993), or culture (Soenens et al., 2015). In other words, it has been implicitly assumed that families that share the same *group-differential* characteristics tend to be influenced in quite similar ways. On the other hand, other theoretical accounts have adopted an *idiosyncratic* view, suggesting that how parents and adolescents influence each other is unique to each family (Granic et al., 2008; Grusec, 2008). For example, according to ecological models, the nature of parent-adolescent dynamics varies not only due to the characteristics of the developing adolescent, but also because of the changing characteristics of the context and timing of events (Bronfenbrenner, 2005; Sameroff, 2010). To date, it has not yet been empirically determined whether subgroups of families function similarly or whether each family functions in their own idiosyncratic way. The primary reason why is that it has rarely been tested how parents and adolescents influence each other in a diverse range of behaviors and emotions at the level of the individual family (for reviews see Boele et al., 2020; Keijsers et al., 2022). To advance empirical knowledge, this 100-day diary study examined the daily dynamics between various parenting practices and adolescent affect in individual families and explored whether data-driven subgroups of families emerged that exhibited similar dynamics.

### The Study of Subgroups with Stable Parenting Styles

Previous research has extensively examined the role of parenting styles in explaining differences in the way parents raise adolescents. Parenting styles represent typologies based on combined parenting dimensions, with a focus on the two dimensions of parental support and behavioral control (Baumrind, 1991). Studies have provided valuable insights into how average levels of combined parenting dimensions and adolescent outcomes differ between subgroups of families. For example, adolescents raised by authoritative parents (i.e., high in both support and control) display better psychosocial functioning than those raised by parents with different parenting styles (e.g., Kuppens & Ceulemans, 2019). However, it has been increasingly stressed that group-level (between-family) associations convey little to no information on the dynamic processes that unfold *within families*, that is, how parents and adolescents of the same family influence each other over time (Hamaker, 2012; Molenaar & Campbell, 2009). Accordingly, it remains an open question whether families also differ in their everyday parent-adolescent dynamics, including different kind of parenting behaviors. As everyday influences between parents and their children are believed to be “the primary engines of development” (Bronfenbrenner, 2005, p. 6), it is vital to understand how daily parent-adolescent dynamics unfold (differently) within families.

### Towards Studying Heterogeneous Parent-Adolescent Dynamics in Everyday Life

**Fig. 1:**
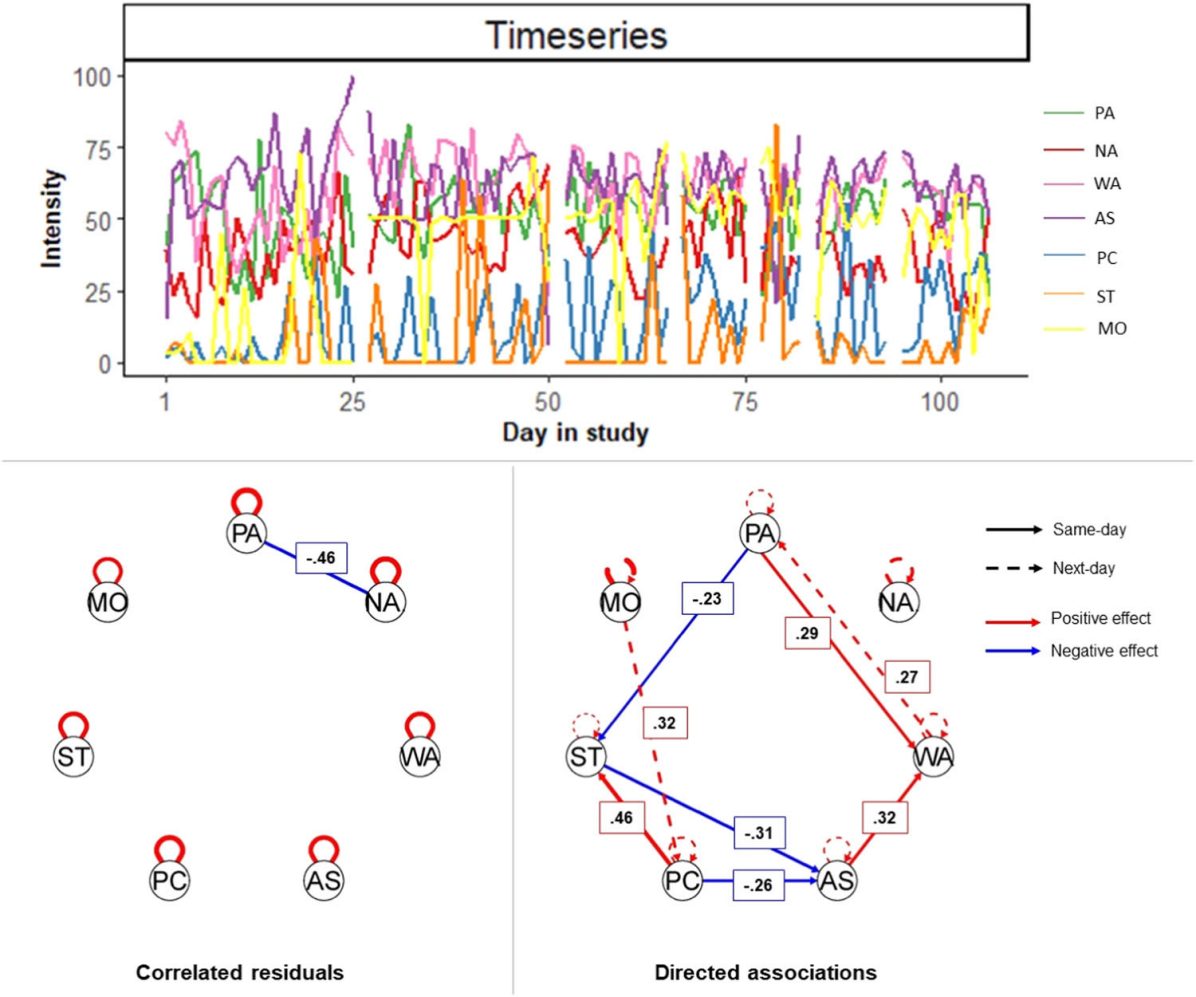
Example of family-specific time series and corresponding temporal network model. *Note*. The network includes (clockwise direction) adolescent positive (PA) and negative affect (NA), parental warmth (WA), autonomy support (AS), psychological control (PC), strictness (ST), and monitoring (MO). Line thickness reflects the magnitude of the association. Beta are displayed in boxes. Hybrid-GIMME allowed to model undirected same-day associations (i.e., correlated residuals) and directed same- or next-day associations. Family-specific model fit: χ^2^(54) = 639.50, *p* = 0.167, RMSEA = 0.04, SRMR = 0.07, NNFI = 0.94, CFI = 0.97

In addition to more stable styles, parents express a wide range of more specific behaviors, also called practices (e.g., warmth, strictness), which fluctuate across time and situations (Darling & Steinberg, 1993). Fluctuations in parenting practices are believed to have a direct impact on adolescent well-being (Darling & Steinberg, 1993) and vice versa (Kuczynski & Parkin, 2007). Such daily influences between parents and adolescents are believed to vary across (subgroups of) families, due to individual factors (e.g., personality; Pluess, 2015), contextual factors (e.g., culture; Soenens et al., 2015), or a family’s unique interplay between various factors at multiple levels (Bronfenbrenner, 2005; Van Geert & Lichtwarck-Aschoff, 2005). Hence, to understand how parenting practices affect the everyday well-being of adolescents, it is vital to investigate (a) how daily fluctuations in the two are associated within individual families (see Fig. 1 for an example of such daily fluctuations) and (b) how such associations differ across these individual families.

With the increasing technological possibility of collecting intensive longitudinal data (e.g., experience sampling and daily diary), an increasing number of intensive longitudinal studies on parenting adolescents are being conducted (for a review see Boele et al., 2020). These studies have provided several insights. First, different kinds of parenting practices indeed fluctuate across time, such as from moment to moment or day to day (for example, see Fig. 1). Second, such over-time fluctuations in parenting practices are associated with fluctuations in adolescents’ well-being within “the average family” (Boele et al., 2020). For example, more daily parental warmth than typical is related, *on average*, to a more positive affect in adolescents (e.g., Bülow, Neubauer, et al., 2022; Xu & Zheng, 2022). Third, initial studies suggest, however, that daily parenting effects within the average family are unlikely to apply to each and every individual family. That is, the nature of the bivariate associations between parenting and adolescent functioning and well-being has been found to vary substantially across families (e.g., Keijsers et al., 2016; Mastrotheodoros et al., 2022). For instance, some adolescents (more than others) show improved well-being when experiencing more parental warmth (Bülow, Neubauer, et al., 2022; Bülow, Van Roekel, et al., 2022), whereas others show worse well-being (Boele et al., 2023; Janssen et al., 2021). Thus, recent work has provided the first evidence that daily influences between parenting and adolescents’ well-being differ across families. The next step is to untangle how the effects of diverse parenting practices converge within a family and how families differ therein, and to what extent.

Various studies have tried to illuminate why families differ in their everyday parenting effects, with some studies detecting meaningful differences, whereas others did not (Boele et al., 2020). For instance, one study found that daily increases in parent-child conflict and adolescent emotional distress were more strongly linked in female adolescents than in male adolescents (Chung et al., 2009); while other studies found no sex differences (e.g., Janssen et al., 2021; Timmons & Margolin, 2015). Daily parent-child conflict and parental support co-fluctuated more strongly with negative affect among adolescents with more depressive and anxiety symptoms (Janssen et al., 2021; Timmons & Margolin, 2015), and higher trait levels of environmental sensitivity and neuroticism in adolescents have been linked to stronger same-day associations between parental support and adolescent affect (Bülow, Neubauer, et al., 2022). Hence, empirical studies suggest that adolescent characteristics such as sex, psychological functioning, and personality may contribute to differences across families in how parents and adolescents influence each other in everyday life.

### Advancing the Field: A Bottom-Up Idiographic Network Approach

While recent studies have provided valuable empirical insights, prior studies bear two limitations. A first key limitation is the wide application of a top-down approach. That is, studies have typically clustered families into pre-determined subgroups based on trait-like characteristics such as adolescents’ sex, and subsequently, assessed whether these subgroups show average differences in bivariate associations between parenting and adolescent well-being (i.e., aggregate, *then* analyze; e.g., Chung et al., 2009). Hence, much of previous research has operated under the assumption that families placed within the same subgroup function quite similarly. To address this limitation, a novel approach can be adopted: a bottom-up approach. With a bottom-up approach, it can be tested whether families can be grouped based on similarities in their parent-adolescent dynamics (i.e., analyze, *then* aggregate), for example by using data-driven subgrouping procedures (Gates et al., 2017).

Another limitation of prior research is the focus on how families differ in *bivariate* associations between parenting and adolescent well-being. As the parenting style literature emphasizes (Baumrind, 1991), parenting adolescents entails various practices, which may even be displayed at the same time and may influence each other (Darling & Steinberg, 1993). Moreover, underlying individual and contextual characteristics might define the entire nature of a family’s parent-adolescent dynamics. For instance, some adolescents may be more responsive to both positive and negative parenting practices because of their general heightened sensitivity to the environment (Pluess, 2015). As such, it is crucial to explore how multiple parenting practices are intertwined with an adolescent’s well-being, for example by adopting a dynamic network approach (Beltz & Gates, 2017), and to explore how such *patterns* of associations vary among families.

To overcome aforementioned limitations and enhance the empirical understanding of parenting adolescents, the current study applied Group Iterative Multiple Model Estimation (GIMME; Gates et al., 2017). GIMME is a data-driven method for estimating idiographic (in this application, family-specific) dynamic network models of contemporaneous and lagged directed associations among the many included variables. Here, same-day and next-day associations were estimated among five parenting practices (i.e., warmth, autonomy support, psychological control, strictness, and monitoring; Smetana, 2017), which are widely regarded as universally influential in shaping adolescents’ well-being (Soenens et al., 2017), and two dimensions of adolescent affective well-being (negative and positive affect; Diener et al., 2018). A visualization of a family-specific network model is shown in Fig. 1, including the underlying time-series data. The Subgrouping GIMME algorithm can additionally detect whether the temporal associations in the family-specific networks are shared by the entire sample, shared by a specific subgroup, or are unique to an individual family (Lane et al., 2019).

## The Current Study

Although numerous theories suggest that parents and adolescents influence each other in diverse ways, valid empirical evidence is still needed to determine the degree to which these influences vary across families. Therefore, the main aim of the current study was to examine whether daily parent-adolescent dynamics are shared by subgroups of families (i.e., group-differential) or are unique to each family (i.e., idiosyncratic). To achieve this, this family-specific dynamic network study investigated how five key parenting practices interplayed with adolescents’ affect in each family’s everyday life, and whether data-driven subgroups of families exhibited similar patterns of associations. If subgroups emerged, an additional aim was to identify adolescent attributes that potentially explained differences between families of different subgroups (i.e., average levels of daily parenting and affect, adolescent psychological functioning, demographic characteristics, legitimacy beliefs of parental authority, and personality traits).

## Methods

### Participants

A total of 159 adolescent-parent dyads participated in the 100-day diary study “100 days of my life” (https://osf.io/5mhgk/; Bülow, Neubauer, et al., 2022). Adolescents were included in the current study if they completed at least 80 daily diaries and showed variance in all the included daily variables, leading to a final sample of 129 adolescents (*M*_age_ = 13.3 years old; *SD*_age_ = 1.2, range 12–16). Of these 129 adolescents, 64% were female (36% male, 2% identified as neither male nor female), and most were born in the Netherlands (87%). A minority were born in other European countries (6%), or counties in Asia (2%), North America (1%), South America (1%), or Africa (1%). Their educational levels varied between pre-vocational secondary education or vocational training (14%), higher general secondary education (29%), and pre-university secondary education (51%). Some of the participants followed a mixed educational track (5%). Moreover, 55% of the adolescents reported to be nonreligious and not baptized, 22% reported to be nonreligious but baptized, and 22% reported being religious, with most affiliating with Christianity (93%). The majority of adolescents lived together with both of their parents (74%) and a minority lived with both parents but in different homes (19%) or reported other living situations (e.g., living only with mother). Almost all the adolescents had at least one sibling (95%), with the majority having one (50%) or two siblings (35%). Among these adolescents with siblings, the distribution of birth order was as follows: 52% were the eldest, 32% were the second child, 15% were the third, 1% were the fourth, and 1% were the fifth.

Adolescents reported on one participating primary caregiver of choice: biological mother (78%), biological father (20%), or other (*n* = 1 adoption mother or *n* = 1 other mother). The parents were on average 45.2 years old (*SD* = 4.59, range = 33–55). Most were born in the Netherlands (91%) and a minority in other European countries (5%), Asia (2%), North America (1%), and Africa (1%). Additionally, 12% of the parents only completed high school, and 26% completed vocational/technical training and 59% college or university, and 2% provided insufficient information. The majority of the parents reported to be nonreligious (60%). Parents who reported to be religious mostly affiliated with Christianity (86%).

### Procedure

Most parent-adolescent dyads were recruited via two high schools in the Netherlands, which offered all secondary educational tracks to 1,300 and 2,000 students, respectively. Adolescents and their parents were informed by class visits, email, and posters. Interested families received a detailed briefing via a video call, after which they received online informed consent forms. Parents also provided informed consent for the participation of their underaged adolescent. One dyad (i.e., composed of an adolescent between 12 – 16 years old and one parent with whom they had daily contact) could participate per family. Both members of the dyad needed to own a smartphone in order to participate. When multiple children in a household were eligible, the family themselves could decide who would participate in the study.

For 100 consecutive days (October 26, 2020, until February 2, 2021), adolescents received daily surveys (ca. 3–5 min) via the Ethica Data smartphone app. The surveys were prompted between 7 PM and 10 PM, depending on their preference. A maximum of four automatic reminders were sent in the evening and one final reminder at 7AM the following morning. To ensure compliance, several motivational features were added. Specifically, adolescents received a monetary reward for each completed survey and bonus if they completed 10 surveys in a row and 100 surveys in total. Overall, adolescents could receive up to €100 (approximately US$121), and €10 was raffled off daily to two adolescents who completed the daily survey. Missed surveys could be compensated by extending the participation period to a maximum of 25 days. This resulted in an average of 93 completed (*SD* = 15.7, range = 24 – 108) and 13 missing daily diaries (*SD* = 16.6, range = 0–76) per person, with most completed in the evening (80%). Because adolescents did not speak to their parents on all days, an average of 91 daily parenting reports were obtained (*SD* = 16.2, range = 24–108). This study was approved by the Ethical Committee of Tilburg University (RP250). More detailed information about the procedure can be found online: https://osf.io/5mhgk/. Parts of the data were analyzed in prior work (Boele, Bülow, Beltz, et al., 2023; Bülow, Neubauer, et al., 2022; De Vries et al., 2023).

### Measures

All daily diary items were scored on a visual analog scale (VAS) ranging from 0 (*Not at all*) to 100 (*Very much*).

### Parenting practices

#### Warmth

Parental warmth includes (a) provision of affection and (b) parental care and responsiveness (Soenens et al., 2017), which were rated by adolescents with two items. The two items were adapted from a Dutch daily diary study (Keijsers et al., 2016), which was, in turn, based on the widely used Network of Relationships Inventory (NRI; Furman & Buhrmester, 1985). The items were: “The relationship with my parent was enjoyable” and “My parent showed me that she/he cares for me.” The internal consistency of the two items was acceptable at the within-family level (*r* = 0.63, *p* < 0.001) and good at the between-family level (*r* = 0.83, *p* < 0.001). The 100-day average of daily warmth was strongly correlated (*r* = 0.60, *p* < 0.001) with a support/warmth subscale of the well-established Network Relationship Inventory (NRI), with the latter measured once during the study (for more information about the study design, see https://osf.io/5mhgk/), providing evidence of convergent validity for the novel daily parental warmth scale.

#### Autonomy support

Parental autonomy support is defined by (a) the provision of choice and allowance of independent decision-making and (b) acknowledgment and interest in the adolescents’ perspective (Soenens et al., 2017). To capture both components, two items were used that were adapted from a 4-item daily autonomy support scale (van der Kaap-Deeder et al., 2017), which was based on the Perception of Parents Scale (POPS; Grolnick et al., 1991). The items were “My parent allowed me to make my own plans” (independent decision-making) and “My parent took my point of view into account” (acknowledgment of perspective). Internal consistency of the 2-item scale was good at both the within-family (*r* = 0.45, *p* < 0.001) and between-family level (*r* = 0.71, *p* < 0.001), indicated by moderate to strong inter-item correlations. The 100-day average of daily autonomy support was strongly correlated (*r* = 0.67, *p* < 0.001) with a once measured POPS subscale (more information about the study design, see https://osf.io/5mhgk/), suggesting convergent validity for the daily parental autonomy support scale.

#### Psychological control

Psychological control involves regulating children’s thoughts and emotions through manipulative behaviors, including (a) constraining verbal expression, (b) guilt induction, and (c) love withdrawal (Barber, 1996). To measure these parenting behaviors, three items adapted from an existing 4-item daily diary scale were used (van der Kaap-Deeder et al., 2017), which was in turn based on the widely used Psychological Control Scale (Barber, 1996). The items were: “When I wanted to say something, my parent started to talk about something else” (constraining verbal expressions), “My parent blamed me for the problems at home” (guilt induction), and “My parent was less affectionate towards me when I did not see things his/her way” (love withdrawal). Multilevel confirmatory factor analysis (Geldhof et al., 2014) indicated moderate reliability at the within-family level (ω = 0.59) and good reliability at the between-family level (ω = 0.83). Regarding convergent validity, the 100-day average of daily psychological control was strongly correlated (*r* = 0.53, *p* < 0.001) with the established Psychological Control-Disrespect Scale (Barber et al., 2012) that was measured once during the study.

#### Strictness

Parental strictness and rule setting are aimed at controlling the behavior of their adolescent children (Kerr et al., 2012). The current study measured this with one item: “My parent was strict.” This item was adapted from a previous work (Stattin & Kerr, 2000). The 1-item measures of daily strictness and monitoring correlated weakly across days within persons (*r* = 0.10, *p* < 0.001), which indicates that the items might indeed have measured different parenting practices. Hence, although strictness and monitoring are both components of the parenting dimension ‘behavioral control’ (Smetana, 2017), the low correlation suggests the necessity of distinguishing these practices in daily life.

#### Monitoring

In addition to strictness, parents can actively monitor their adolescents’ whereabouts and activities to control their behavior (Kerr et al., 2012). To measure parental monitoring, adolescents responded to the following item: “I had to tell my parent what I did, with whom, and where.” This item was adapted from a parental monitoring questionnaire (Stattin & Kerr, 2000). The 100-day average of the single-item monitoring scale correlated moderately (*r* = 0.39, *p* < 0.001) with the behavioral control scale of a commonly used questionnaire (Stattin & Kerr, 2000) that was measured once.

### Adolescent affective well-being

Affective well-being can be defined as high levels of positive affect (i.e., pleasant and desirable feelings) and low levels of negative affect (i.e., unpleasant and undesirable feelings) (Diener et al., 2018). To measure daily affective well-being, five items from the Positive and Negative Affect Schedule for Children were used (PANAS-C) (Ebesutani et al., 2012), which were chosen based on the psychometric properties of the Dutch scale in an adolescent sample in a previous study (Bülow, Van Roekel, et al., 2022). That is, positive affect was measured with two items (“joyful” and “happy”), and negative affect with three items (“mad”, “afraid”, and “sad”). Internal consistency of the 2-item positive affect scale was good at the within-family level (*r* = 0.75, *p* < 0.001) and excellent at the between-family level (*r* = 0.95, *p* < 0.001). Similarly, the internal consistency of the 3-item negative affect scale was good at the within-family level (ω = 0.71) and excellent at the between-family level (ω = 0.92).

### Pre-registered analytical approach

#### S-GIMME

To answer the research question whether subgroups of families exist who share similar daily parent-adolescent dynamics, a pre-registered (see https://osf.io/a4rzm/) Subgrouping Group Iterative Multiple Model Estimation (S-GIMME; Gates et al., 2017; Lane et al., 2019) was conducted, by using the R package *gimme* version 0.7–10 (Gates & Molenaar, 2012). Figure 1 visualizes how this study used GIMME, a data-driven statistical technique, to estimate sparse unit-specific (here: family-specific) temporal networks. GIMME is particularly well-suited for estimating the heterogeneity of associations in intensive longitudinal data from heterogeneous samples (Gates & Molenaar, 2012).

**Fig. 2:**
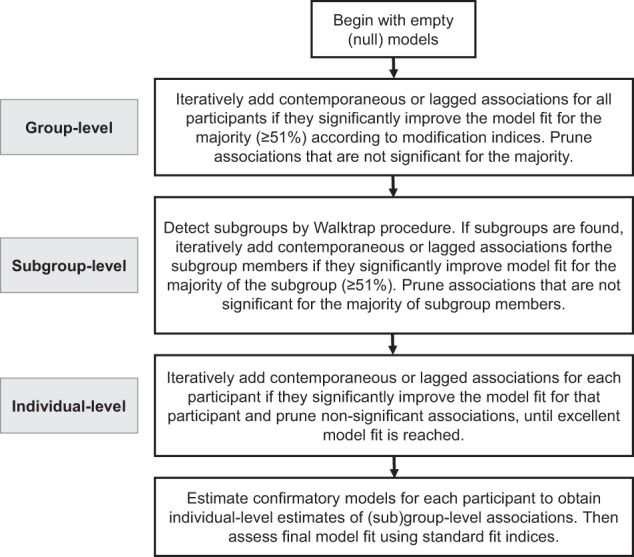
Summary of model fitting with the S-GIMME algorithm. *Note*. In the current study, same-day (contemporaneous) and next-day (lagged) associations were estimated among five parenting practices and two adolescents affect network nodes

To estimate a family-specific network, as well as (sub)groups of similarly functioning families, GIMME implements family-specific unified structural equation models (see Gates et al., 2010). These models are a type of structural vector autoregressive (VAR) model that combines traditional VAR and structural equation models (SEM) to simultaneously estimate directed lagged (i.e., first-order next-day) and contemporaneous (i.e., same-day) associations. GIMME implements these family-specific models within a grouping algorithm that prioritizes the estimation of relationships that are common across participants (if any exist). All technical steps are summarized in Fig. 2. GIMME handles missing data using full information maximum likelihood (FIML; Beltz & Gates, 2017).

To achieve this, GIMME begins with empty “null” network models. Then, *group-level associations* are iteratively added to all empty family-specific networks if they significantly (after Bonferroni correction of 0.05/*N*, thus the alpha level here was 0.004) improve the model fit for the majority of the sample (here, 51%; see Gates et al., 2020) according to Lagrange multiplier equivalence tests (i.e., modification indices; Sörbom, 1989). To improve path recovery, autoregressive effects were estimated for every family-specific network, by default (Lane et al., 2019). Subsequently, the *subgrouping option* within the GIMME algorithm clusters individual families using Walktrap community detection, based on similarities in family-specific estimates of (1) group-level associations and (2) associations that are likely to emerge at the individual family level. Subgroup-level associations are iteratively added to the family-specific networks of the subgroup members if they significantly (Bonferroni corrected) improve the model fit for the majority of the subgroup members (again 51%; Gates et al., 2017; Lane et al., 2019) according to modification indices. Finally, *individual-level associations* (i.e., associations unique to each family) are iteratively added to a family’s network by evaluating whether they significantly (*p* < 0.01) improve model fit. At the group, subgroup, and individual levels, iterations stop if the model fits well (i.e., if two of the four criteria are met: RMSEA ≤ 0.05, SRMR ≤ 0.05, CFI ≥ 0.95, and NNFI ≥ 0.95). The model will also be pruned, with non-significant associations. Hence, S-GIMME allowed us to identify general (sample-level), subgroup-specific (subgroup-level), and family-specific (individual-level) associations, all of which had family-specific magnitudes.

Notably, the hybrid-GIMME option was used to enable the estimation of data-driven undirected contemporaneous associations that likely exist because of a shared exogenous influence (Luo et al., 2022). These *undirected* contemporaneous (here same-day) associations reflect the correlations among the variable residuals. They are ideal for mapping relations among variables that share measure or method variance (e.g., positive and negative affect or associations among parenting practices).

#### Explaining differences between subgroups and individual families

In the second step of the pre-registered analysis, differences between subgroups were explored in terms of personal and family characteristics. That is, subgroups were compared based on several variables: average levels of parenting and affect variables, indicators of adolescent psychological functioning (i.e., depressive and anxiety symptoms, and self-esteem), adolescent demographic characteristics (i.e., age, sex, educational level), legitimacy beliefs about parental authority, and personality traits (i.e., environmental sensitivity, neuroticism). More information about these measures is provided in the online Supplementary Information. To test for group differences in these variables, *t*-tests (with continuous variables, e.g., age) and chi-square tests (with categorical variables, e.g., sex) were conducted.

If no subgroups were identified that shared similar parent-adolescent dynamics, it was pre-registered to describe differences between individual families instead. Specifically, the correlation between the density of parenting-affect associations and the variables described above was calculated. Density was calculated for each family by dividing the number of parenting-affect associations by the total number of associations in their family-specific network model (excluding autoregressive effects). Parenting-affect density thus reflects the extent to which temporal associations between perceived parenting and adolescent affect contribute to the overall family-specific network.

## Results

### Intraclass and Bivariate Correlations

To assess the extent to which parenting practices and affect fluctuated from day to day, intraclass correlation coefficients were calculated (ICCs; see Fig. 1 for an example of the data). The ICCs ranged between 0.49 and 0.64 (see Table 1). In other words, 49–64% of the variance in perceived parenting practices and adolescent affective well-being was due to stable differences between families. Day-to-day fluctuations within families accounted for the remaining 36%–51% of the variance.

**Table 1:** Descriptive statistics and correlations (N = 129)

	Descriptive statistics			Correlations						
	*M*	*SD*	ICC	1.	2.	3.	4.	5.	6.	7.
Parenting dimension										
1. Warmth	83.34	17.02	0.56	–	0.39***	−0.33***	−0.30***	0.00	0.32***	−0.23***
2. Autonomy support	74.61	23.99	0.55	0.64***	–	−0.26***	−0.26***	0.07**	0.18***	−0.13***
3. Psy. control	6.77	11.93	0.62	−0.42***	−0.41***	–	0.42***	0.06*	−0.12***	0.16***
4. Strictness	13.08	21.33	0.60	−0.37***	−0.37***	0.64***	–	0.10***	−0.11***	0.16***
5. Monitoring	21.61	29.76	0.59	−0.16*	−0.13	0.28***	0.52***	–	0.06**	0.00
Adolescent affect										
6. Positive affect	76.27	20.93	0.64	0.46***	0.39**	−0.11	−0.19*	−0.09	–	−0.50***
7. Negative affect	11.11	15.17	0.49	−0.36***	−0.33***	0.39***	0.49***	0.20*	−0.66***	–

All the items ranged from 0 to 100. Within-family correlations are presented above the diagonal, and between-family correlations are presented under the diagonal. * *p* < 0.05, ** *p* < 0.01, *** *p* ≤ 0.001

*M* sample mean, *SD* standard deviation, *ICC* intraclass correlation coefficient, *Psy. control* psychological control

Descriptive statistics and bivariate correlations among parenting and adolescent affect variables are provided in Table 1. Perceived parenting practices and adolescents’ affective well-being were weakly to moderately correlated at the within-family level. On average, adolescents perceived more parental warmth and autonomy support on days they also experienced more positive affect (*r*s ≥ 0.18, *p* < 0.001) and less negative affect (*r*s ≤ −0.13, *p* < 0.001). More parental psychological control and strictness correlated with less positive affect (*r*s ≤ −0.11, *ps* < 0.001) and more negative affect (*r*s = 0.16, *ps* < 0.001) within families. More parental monitoring correlated with more same-day positive affect (*r* = 0.06, *p* = 0.003) but not with less negative affect (*r* = 0.00, *p* = 0.823). Compared to these average within-family correlations, between-family correlations were similar in sign, but stronger in magnitude.

### Family-Specific Temporal Network Models

Rather than focusing on the average within-family associations in this study, it was examined how individual families function. The data were well-suited to assess these dynamics: 127 of 129 family-specific network models showed excellent model fit, according to the mean RMSEA = 0.04 (range:0.00–0.10), SRMR = 0.07 (range: 0.04–0.15), CFI = 0.97 (range:0.95–1.00), and NNFI = 0.99 (range:0.92–1.00). Two family-specific networks that failed to achieve a satisfactory model fit (RMSEA ≥ 0.11, SRMR ≥ 0.08, CFIs ≤ 0.93, NNFI ≤ 0.88) were removed from the subsequent analyses.

#### Associations shared by all families (group level)

Before answering the research question about the existence of subgroup dynamics, it was explored whether ‘group-level’ associations existed, i.e., those that were estimated for all families. One data-driven group-level association existed, which was an exogenous association between adolescent positive and negative affect (see Fig. 2). For most adolescents (*n* = 116, 91%), increased positive affect co-fluctuated with decreased negative affect across days. In 11 adolescents (9%), increased positive affect co-fluctuated with *increased* negative affect across days. No same-day or next-day associations between parenting practices and adolescent affective well-being were found at the group level (i.e., shared by the majority of families). The current data thus do not provide evidence of the existence of ‘general’ parent-adolescent dynamics in daily life.

#### Associations shared by subgroups (subgroup level)

**Fig. 3:**
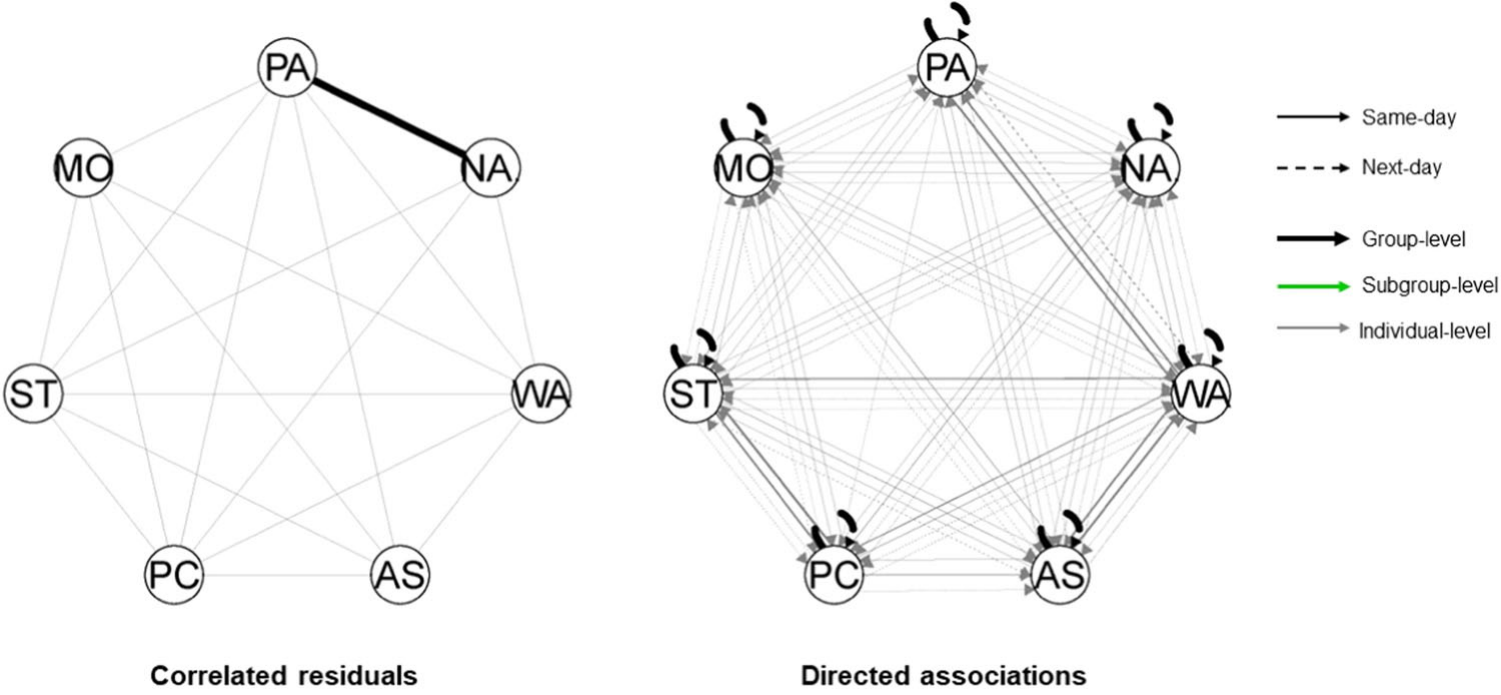
Summary plots of Subgroup 1 (*n* = 77). *Note*. Hybrid-GIMME allowed to model undirected same-day associations (left) and directed same- or next-day associations (right). The black line between adolescent positive and negative affect in the left figure is a same-day association estimated for everyone in the sample, and thus, also in this subgroup. No subgroup-specific associations were found; therefore, no green lines were depicted in either figure. The gray lines in both figures are individual-level associations found for one or more individual families in this subgroup, with line thickness corresponding to the number of families for which that association was estimated. The arrows indicate the directionality of the association. PA positive affect, NA negative affect, WA warmth, AS autonomy support. PC psychological control, ST strictness, MO monitoring

**Fig. 4:**
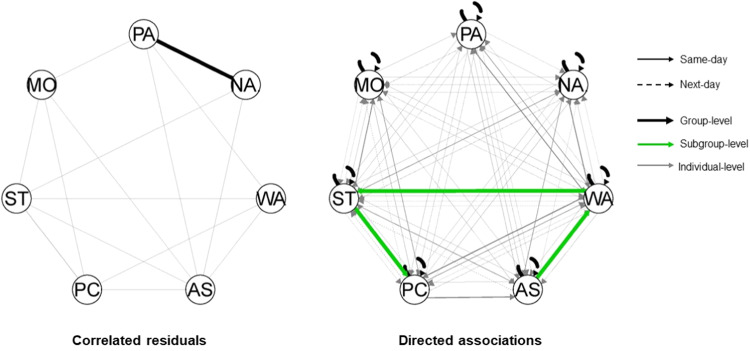
Summary plots of Subgroup 2 (*n* = 45). *Note*. Hybrid-GIMME allowed to model undirected same-day associations (left figure) and directed same- or next-day associations (right). On the left: The black line between adolescent positive and negative affect is a same-day association estimated for everyone in the sample and, thus, also in this subgroup. On the right, the green lines represent the subgroup-specific associations estimated for everyone in this subgroup. The gray lines in both figures are individual-level associations found for one or more individual families in this subgroup, with line thickness corresponding to the number of families for which that association was estimated. The arrows indicate the directionality of the association. PA positive affect. NA negative affect, WA warmth. AS autonomy support. PC psychological control, ST strictness, MO monitoring

Seven data-driven subgroups were identified. The two subgroups were comprised of 77 and 45 families, respectively. Families in Subgroup 1 (*n* = 77; see Fig. 3) had no subgroup-specific associations and thus only shared a group-level association between adolescent positive and negative affect, with 70 families showing a negative association. Families in Subgroup 2 (*n* = 45; see Fig. 4) showed three subgroup-specific same-day parenting-to-parenting associations: strictness → psychological control (positive effect: *n* = 41, negative effect: *n* = 4), warmth → autonomy support (positive effect: *n* = 42, negative effect: *n* = 3), and warmth → strictness (positive effect, *n* = 43; negative effect, *n* = 2).

The other five subgroups were singletons, each of which was placed in a subgroup by themselves. Thus, these five families deviated from the sample in terms of their temporal association patterns (Lane et al., 2019), indicating that their dynamics were particularly unique. The family-specific networks of these five families can be viewed in the online Supplementary Information (see Fig. S1).

#### Associations unique to families (individual level)

Although no parenting-affect associations were detected for the full sample or subsample, many unique associations were found within individual families. In fact, 109 (86%) of the 127 family-specific network models contained at least one significant (*p* < 0.05) association between one of the five parenting practices and adolescent positive or negative affect. Of the 109 families, 51 families had only same-day associations, eight families had only next-day associations, and 50 families had same-day as well as next-day associations. Of the total 264 estimated parenting-affect associations, 179 (68%) were same-day associations, of which 154 were directed, 25 were undirected (i.e., correlated residuals), and 85 (32%) were next-day associations. On average, the families displayed 2.4 parenting-affect associations (*SD* = 1.3, range = 1–7). Parenting-affect associations accounted for 9% to 75% of the total associations in the family-specific networks (excluding autoregressive effects).

To provide more in-depth insights into these family-specific dynamics, the parenting-affect associations found at the individual level were elaborated upon. As summarized in Table 2, each of the five parenting practices was associated with adolescents’ affective well-being across families. However, which parenting practices were related to adolescent positive or negative affect, how strong, and at which timescale (i.e., same-day and/or next-day) were heterogeneous across families. To illustrate, the association shared by the greatest number of families was a positive same-day association between parental warmth and adolescent positive affect, found in 49 families. All other parenting-affect associations were shared by a maximum of 17 families, with several associations detected in only a handful of families. Thus, although parenting practices were associated with adolescent affective well-being in almost all families, *which* practices were associated with the adolescent’s affective well-being was family specific.

**Table 2:** Number of Families with Same-day and Next-day Associations between Parenting Practices and Adolescent Affect

	Same-day		Next-day	
	*–*	+	*–*	+
Warmth with PA	2	49	1	14
Warmth with NA	25	1	3	5
Autonomy support with PA	0	17	2	7
Autonomy support with NA	11	3	3	4
Psychological control with PA	2	1	2	3
Psychological control with NA	2	12	3	7
Strictness with PA	8	8	4	2
Strictness with NA	2	21	3	4
Monitoring with PA	2	7	8	3
Monitoring with NA	2	5	2	5

*Note*. - = negative association. + = positive association. PA = positive affect. NA = negative affect. All same-day and next-day associations were directed from parenting to affect, or from affect to parenting. Some families were counted twice if they had reciprocal same-day or next-day associations (e.g., warmth predicted PA on the same day *and* vice versa).

**Fig. 5:**
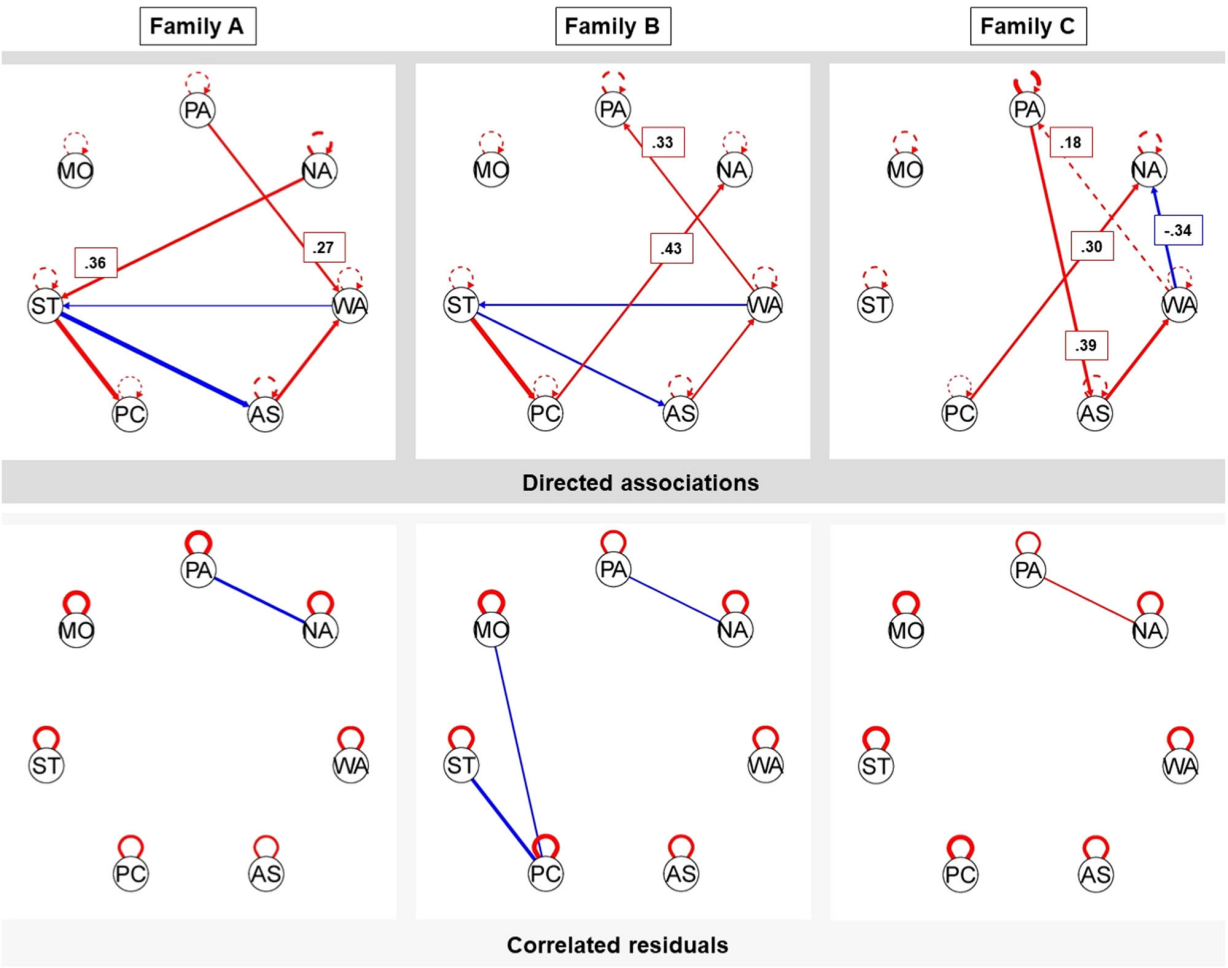
Network Plots of Three Heterogeneous Families. *Note*. Hybrid-GIMME allowed to model undirected contemporaneous associations (lower figures) and directed contemporaneous or lagged associations (upper figures). Solid lines reflect contemporaneous (same-day) associations, dashed lines reflect lagged (next-day) associations, red lines reflect positive associations, and blue lines reflect negative associations. Betas are provided for the parenting-affect associations. Model fit: Family A (χ^2^(56) = 661.12, *p* = 0.167, RMSEA = 0.04, SRMR = 0.07, NNFI = 0.96, CFI = 0.98), Family B (χ^2^(54) = 674.13, *p* = 0.104, RMSEA = 0.05, SRMR = 0.08, NNFI = 0.93, CFI = 0.96), Family C (χ^2^(57) = 661.18, *p* = 0.191, RMSEA = 0.04, SRMR = 0.07, NNFI = 0.94, CFI = 0.97). PA positive affect, NA negative affect, WA warmth, AS autonomy support, PC psychological control, ST strictness, MO monitoring

To further illustrate the idiographic nature of the daily dynamics between parenting practices and adolescents’ affective well-being, Fig. 5 depicts the temporal networks of three individual families. In Family A, more positive affect predicted more parental warmth and more negative affect predicted more parental strictness on the same day. In Family B, more parental warmth predicted more same-day positive affect and more parental psychological control predicted more same-day negative affect. Family C showed both same-day and next-day associations. Specifically, more positive affect predicted more parental autonomy support, and more parental psychological control and less parental warmth both predicted more negative affect on the same day. More parental warmth also predicted more positive affect the *next* day. The temporal networks of all 127 families, including the model fit and family-specific path estimates, can be viewed at https://osf.io/a4rzm/.

#### Sensitivity analyses

In the main findings, five family-specific networks showed temporal associations with low or high beta (< −1.0 or > 1.0) and high corresponding standard error (>1.0). To assess the extent to which the data of these five families affected the main findings, S-GIMME was again performed while excluding these five families. Similar to the main findings, daily associations between parenting and adolescent affect were found only at the individual level (i.e., found in one or some families), and thus not at the group or subgroup level. The summary network plots of these sensitivity analyses can be found in the online Supplementary Information (see Figs. S2–S4).

### Explaining Differences between Subgroups and Individual Families

First, it was tested whether families of subgroups 1 and 2 differed from each other in the following pre-registered variables: mean levels of daily parenting and adolescent affect, adolescent psychological functioning (i.e., depressive and anxiety symptoms, and self-esteem), demographic characteristics (i.e., sex, age, and educational level), legitimacy beliefs, and personality traits (i.e., environmental sensitivity and neuroticism). However, no significant group differences were found (see Table S1 in the online Supplementary Information).

Second, because parenting-affect associations were unique to families, it was also explored whether differences between individual families could be explained. However, the above-described variables were not significantly related to parenting-affect density (see Table S2 in the online Supplemental Information), which reflects the extent to which the temporal parenting-affect associations contributed to the overall family-specific network (excluding autoregressive effects). Hence, the means of the daily assessments and adolescent characteristics did not explain why some families demonstrated more daily associations between perceived parenting practices and adolescent affective well-being than other families.

## Discussion

There is a theoretical consensus among human development (e.g., Bronfenbrenner, 2005; Smith & Thelen, 2003) and parenting theories (e.g., Darling & Steinberg, 1993; Granic et al., 2008) that parents and adolescents influence each other heterogeneously across families. However, different ideas have been formulated regarding the expression of heterogeneity. On the one hand, scholars have assumed that some families are like some others: families sharing certain group-differential characteristics (e.g., personality, parenting style, and culture) could show similar dynamics (e.g., Belsky & Pluess, 2009; Darling & Steinberg, 1993; Soenens et al., 2015). On the other hand, scholars have assumed that each family is like no other family: because everyone has unique experiences, shaped by many interacting and dynamic individual and contextual factors, family dynamics are potentially idiosyncratic to every family (e.g., Bronfenbrenner, 2005; Van Geert & Lichtwarck-Aschoff, 2005). Determining whether subgroups of families function similarly or whether each family functions uniquely is crucial for informing future parenting interventions because these empirical insights will help to determine whether tailored or personalized parenting interventions are needed (August & Gewirtz, 2019; Yap et al., 2019).

Aided by a novel research design in which 127 families were followed for 100 consecutive days, the current study examined whether daily parent-adolescent dynamics were either group-differential or idiosyncratic to each family. That is, a data-driven temporal network procedure was applied to examine whether subgroups of families could be identified that share similar temporal (i.e., same-day and next-day) associations between five perceived parenting practices and adolescent positive and negative affect (Gates et al., 2017). The current findings suggest that parenting and adolescent affective well-being were associated in almost all families (86%). However, no data-driven subgroups of families emerged that shared similar parent-adolescent dynamics. Instead, same-day or next-day associations between parenting practices and adolescent affect were found only at the individual family level, meaning that daily parenting-affect associations were specific to only one or a few families.

### Daily Idiosyncratic Parent-Adolescent Dynamics were Observed in Most Families

Theories of human development postulate that parenting is an important proximal factor shaping an individual’s development (Bronfenbrenner, 2005; Sameroff, 2010). Indeed, the current 100-day diary study rigorously demonstrated that perceived parenting practices are linked to adolescents’ affective well-being in almost all families. That is, in the majority of families (109 out of 127), daily associations were identified between perceived parenting practices and adolescent affect. Going beyond previous studies demonstrating daily linkages between parenting and adolescent affect within the ‘average family’ (e.g., Chung et al., 2009; Schacter & Margolin, 2019), the current family-specific study revealed that such daily linkages could also be observed in most individual families.

Despite the omnipresence of daily dynamics between parenting practices and adolescent affect, no evidence was found of general dynamics (i.e., shared by the whole sample). That is, whether and how the five parenting practices were intertwined with the adolescents’ positive and negative affect varied considerably among the families. It is, however, noteworthy that a substantial portion of the adolescents reported feeling more positive (36%) and less negative (20%) on days when they perceived increased parental warmth. This suggests that parental warmth contributes to the daily affective well-being of many adolescents, though not universally.

Furthermore, no subgroup-specific (i.e., shared by a subgroup of families) parent-adolescent dynamics were found. In other words, the nature of daily parent-adolescent dynamics did not generalize to subgroups of families. Although two data-driven subgroups were found (*n*s = 77 and 45), families in these subgroups did not share similar daily associations between parenting and affect. Instead, the first subgroup shared a same-day association between positive and negative affect (which was also shared with other families outside this subgroup) and the second subgroup exhibited similar same-day associations between distinct parenting practices. A potential explanation for the same-day parenting-parenting associations in the latter subgroup might be that these adolescents were less capable of differentiating between distinct practices. Thus, despite group-differential effects have been theorized, for instance, due to shared individual characteristics (e.g., personality, parenting style; Pluess, 2015; Darling & Steinberg, 1993) or contextual characteristics (e.g., culture; Soenens et al., 2015), the current findings do not support the notion that subgroups of families exhibit homogenous parent-adolescent dynamics in everyday life exist.

### Understanding Parenting as an Idiosyncratic Phenomenon

Many theories focus on the unique (subjective) experiences of an individual child, shaped by many interacting individual and contextual factors (e.g., Bronfenbrenner, 2005). Thus far, however, the existing paradigm of group-based patterns (i.e., variation between families) has not allowed scholars to understand the unique dynamics of individual families (i.e., over-time variation within families; Molenaar & Campbell, 2009). By examining how daily fluctuations in perceived parenting practices and adolescent affect were related across 100 days in each single family separately, the current study demonstrated that daily parent-adolescent dynamics were highly idiosyncratic. That is, it depended on the family *which* of the parenting practices (i.e., warmth, autonomy support, psychological control, strictness, and monitoring) were linked to the adolescent’s affective well-being (for example, see Fig. 5). Although all five practices showed associations with adolescent affect across the whole sample, individual families demonstrated on average 2.4 temporal associations between parenting practices on the one hand and adolescent positive or negative affect on the other. For instance, in one family, the adolescent experienced more negative affect when perceiving their parent to be stricter that day, whereas in another family, the adolescent experienced more negative affect when perceiving their parents to be more psychologically controlling (and not when stricter; see Fig. 5).

Additionally, *how* perceived parenting was related to adolescent affective well-being also varied across families. Family-specific associations differed in strength, sign (i.e., positive versus negative), and timescale (i.e., on the same or the following day). For example, increased parental strictness predicted more negative affect on the same day in 10 families, with family-specific effect sizes ranging from 0.26 to 0.63, and increased parental strictness predicted *more* next-day negative affect in two families but *less* next-day negative affect in two other families. Thus, the findings are consistent with the developmental principle of multifinality (Cicchetti & Rogosch, 1996), such that the same parenting practice showed differential effects on adolescent affect. Together, the present study and other idiographic studies in the broader field of psychology (e.g., Bouwmans et al., 2018; Kelly et al., 2020) offer empirical evidence for the widely held assumption that psychological heterogeneity is an inherent and universal characteristic of human functioning (Richters, 2021).

To understand why the nature of daily parent-adolescent dynamics was heterogeneous across families, a variety of moderators were tested. Specifically, it was tested whether the extent to which parenting-affect associations contributed to the overall family-specific network (i.e., parenting-affect density) could be predicted by mean levels of daily parenting and affect, demographic (i.e., age, sex, education), psychological functioning (i.e., depressive and anxiety symptoms, and self-esteem), legitimacy beliefs of parental authority, and personality traits (i.e., environmental sensitivity and neuroticism). However, none of the moderators were significantly related to parenting-affect density. One explanation for the lack of moderator effects might be that it is the complex interplay of numerous characteristics at multiple levels that shape a family’s unique dynamics (Bronfenbrenner, 2005). Future studies should investigate whether family-specific dynamics can be explained by the interplay between a broad range of individual and contextual characteristics.

### Practical Implications

This family-specific study is another demonstration of the methodological concern that between-family patterns, such as research on parenting styles (Kuppens & Ceulemans, 2019), provide little to no information on how single, unique families function (Molenaar & Campbell, 2009). This study provides evidence that adolescents from different families vary in terms of the parenting practices they respond to and how they respond. While further research is needed to determine to what extent such parent-child dynamics are also dissimilar between siblings from the same family, the current findings already hold significant implications for the formulation of general parenting guidelines and for intervention (and prevention) efforts aimed to improve family dynamics and adolescent well-being. That is, universal guidelines and approaches (e.g., all parents are told that they should be more autonomy-supportive to improve their adolescents’ everyday feelings) might not work similarly for every family. Consequently, parenting scholars might want to be careful in providing generic parenting guidelines to the general public. Parents who attempt to adhere to general advice that misaligns with their unique family dynamics and needs could unintentionally jeopardize their adolescent’s well-being and might experience feelings of incompetence and parenting stress when such generic advice is not working for them.

The current findings, as well as studies demonstrating that some families benefit more from parenting programs than others (e.g., Weeland et al., 2023), indicate that tailoring parenting inventions may be an important future direction to improve their efficacy (August & Gewirtz, 2019). Parenting interventions may want to learn from contemporary approaches in clinical psychology and psychiatry (Myin-Germeys et al., 2018), and could, for example, implement self-monitoring by using experience sampling methods or daily diaries (Swendeman et al., 2020). Tracking a dyad’s dynamics in their day-to-day lives can enhance the understanding of the dyad’s idiosyncratic dynamics, including their maladaptive dynamics, which again can be used to tailor the intervention to the specific needs of the dyad. Also tracking parent-adolescent dyads during and after an intervention can be useful in evaluating whether the dyadic dynamics have indeed been changing in the desired direction (Bamberger, 2016).

### Limitations and Future Directions

This pre-registered study applied a novel family-specific network approach to the study of parenting adolescents. Although work is needed to replicate current findings, this study rigorously demonstrated the idiosyncratic nature of how perceived parenting practices were intertwined with adolescent affect over 100 days in 127 individual families. However, this study has several limitations. First, this study may have underestimated the heterogeneity. Although participants were sampled from a rural area and all educational levels were present in the data, the study included more adolescents from relatively well-functioning families drawn from a community sample. To further unravel how parenting and adolescent well-being interact within diverse families, future studies should encompass larger and more diverse samples, including various ethnic backgrounds and psychopathology.

Second, due to the design of the study, which permitted the participation of only one parent-adolescent dyad per family, the findings thus reflect how dyads of different families uniquely interact in daily life. Therefore, it remains an open question whether the dynamics between parenting and adolescent well-being are truly idiosyncratic to each dyad or potentially exhibit common patterns within the same family. Future studies exploring the extent to which parent-adolescent dynamics generalize within families (e.g., siblings interacting with the same parent) could offer a more stringent test whether these dynamics are truly idiosyncratic. Such studies might also provide more insights into the role of shared environmental and genetic factors in shaping the nature of daily parent-adolescent dynamics.

Third, because intensive longitudinal methods are still in their infancy in parenting research (Keijsers et al., 2022), it is likely that the observed within-family fluctuations in this 100-day diary study also include measurement errors (Schuurman & Hamaker, 2019). Although methodological work suggests that 60 observations per family are sufficient for S-GIMME (Lane et al., 2019), larger individual time series might increase the precision of the estimated family-specific effects and identify more true heterogeneity (Hoekstra et al., 2022). Therefore, pursuing even more than 100 observations per family in future intensive longitudinal studies might be required to comprehend idiosyncratic family dynamics.

Fourth, as this study was among the first to apply GIMME to parenting data, the direction of contemporaneous (same-day) associations needs to be interpreted with caution; this is a limitation of ‘standard’ GIMME. GIMME multiple solutions (GIMME-MS) have been developed to more robustly examine the directionality of contemporaneous (and even lagged) associations (Beltz & Molenaar, 2016). However, GIMME-MS cannot yet be combined with the subgrouping option. Hence, future research is also recommended to unravel how the direction of influences between parenting practices and adolescent well-being differs across individual families (e.g., Boele, Bülow, Beltz, et al., 2023). This piece of information about parent-adolescent dynamics – who influences whom – (e.g., is the adolescent mainly reacting on the parent or is the parent mainly reacting on their adolescent child) is important for interventions, as it indicates who should be targeted to enable desired change.

Fifth, although the study examined five key parenting practices and both positive and negative aspects of adolescents’ affect, it may have overlooked other practices or well-being indicators that are also relevant in families’ everyday life. Future research might also want to include other practices, such as overprotection (Van Petegem et al., 2022), and other adolescent well-being indicators, such as loneliness (Soenens et al., 2017), to gain a more comprehensive understanding of how and why parent-adolescent dynamics are (dis)similar across families and to adequately inform future practice.

## Conclusion

In almost every family, adolescent-perceived parenting practices were intertwined with the adolescent’s affective well-being in everyday life. However, the findings revealed no evidence of homogeneity in the nature of these daily parent-adolescent dynamics, either at the sample or subgroup level. Instead, daily parent-adolescent dynamics appeared idiosyncratic: *Which* parenting practices were intertwined with the adolescent’s affective well-being, and *how*, was specific to the family. Although future studies with longer time series per family and larger samples are needed to replicate the idiosyncratic findings demonstrated here, the current results suggest that future translational efforts may benefit from tailoring interventions to the specific dynamics of the family.

### Supplementary Information

Online Supplementary Materials
