# Supplementary material for: Like No Other? A Family-Specific Network Approach to Parenting Adolescents
Source: J Youth Adolesc. 2023 Dec 6;53(4):982–97. doi: 10.1007/s10964-023-01912-5 (PMC10879241; doi:10.1007/s10964-023-01912-5)
Supplement: Supplementary file 1 — Online Supplementary Materials [file 10964_2023_1912_MOESM1_ESM.docx]

**Online Supplementary Materials for:**

**Like No Other? A Family-Specific Network Approach to Parenting Adolescents**

Savannah Boele^1^, Anne Bülow^1^, Adriene M. Beltz^2^, Amaranta de Haan^1^, Jaap J. A. Denissen^3^, Marleen H. M. de Moor^1^ & Loes Keijsers^1^

^1^ Department of Psychology, Education and Child Studies, Erasmus University Rotterdam, the Netherlands

^2^ Department of Psychology, University of Michigan, United States of America

^3^ Department of Developmental Psychology, Utrecht University, the Netherlands

**Supplementary Information 1**

**Differences in Adolescent Characteristics**

Here we provide more information about the adolescent characteristics that were used in the moderation analyses. All below-described characteristics were included in an online questionnaire, distributed through e-mail at 25^th^ of October 2020, at the start of the 100 daily diary study (first diary prompted at 26^th^ of October 2020).

**Depressive symptoms.** Adolescent depressive symptoms were measured with the Reynolds Adolescent Depression Scale Short (RADS-2; Milfont et al., 2008, Reynolds, 2005). The scale consists of 12 items (e.g., “I was sad”), with a response scale ranging from 1 (*almost never*) to 4 (*often*). Adolescents had to reflect whether they experienced these symptoms within the last two weeks. Reliability of the scale was excellent (α = .90).

**Anxiety symptoms.** Adolescent general anxiety symptoms were measured with the General Anxiety subscale of the Screen for Child Anxiety Related Emotional Disorders (SCARED; Birmaher et al., 1997). The scale consists of nine items (e.g., “I was worried about how well I was doing things”), with a response scale ranging from 1 (*never*) to 3 (*often*). Adolescents had to reflect whether they experienced these symptoms within the last two weeks. Reliability was good (α = .88).

**Self-esteem.** Adolescent self-esteem was measured with the Rosenberg Self-Esteem Scale (Rosenberg, 1965). Five of the 10 items were included, based on the highest factor loadings in prior work (Franck et al., 2008). An example items is: “I felt that I'm a person of worth, at least on an equal plane with others”. The response scale ranged from 1 (*totally disagree*) to 5 (*totally agree*). Reliability of this five-item scale was good (α = .79).

**Legitimacy beliefs.** Legitimacy beliefs of parental authority was measured with five items (see Smetana & Daddis, 2002, Smetana et al., 2006). The items were: It is okay that my parents make rules about .. (1) the movies and music I watch and listen, (2) what I do in my free time, (3) what I do at someone’s home if there are no adults around, (4), what I may or may not do with friend, (5) with whom I may or may not be friends. The response scale ranged from 1 (*disagree*) to 3 (*totally agree*). Higher scores thus indicated greater legitimacy of parental authority. Reliability of the scale was good (α = .76).

**Environmental sensitivity.** Trait environmental sensitivity was measured with the Hypersensitivity Child Scale (HSC) Short version (Pluess et al., 2018). The short version consists of 12 items (e.g., “I notice when small things have changed in my environment”), with a response scale ranging from 1 (*not at all*) to 7 (*extreme*). Reliability of the scale was good (α = .74).

**Neuroticism.** Trait neuroticism was measured with the Neuroticism subscale of the Big Five Inventory II (S-version; Denissen et al., 2020). The subscale consists of six items, such as “I worry a lot”, which had a response scale ranging from 1 (*totally disagree*) to 5 (*totally agree*). Reliability of the Neuroticism scale was good (α = .79).

**Tabel S1**

*Comparing Families from Subgroup 1 and 2*

|  | *d /* Cramer’s *V* |
| --- | --- |
| ***Average levels of parenting*** |  |
| Warmth | -.17 |
| Autonomy support | -.20 |
| Psychological control | -.24 |
| Strictness | -.08 |
| Monitoring | -.08 |
| ***Average levels of affect*** |  |
| Positive affect | -.15 |
| Negative affect | -.21 |
| ***Adolescent psychological functioning*** |  |
| Depressive symptoms | .07 |
| Anxiety symptoms | .02 |
| Self-esteem | -.03 |
| ***Adolescent demographics*** |  |
| Age | -.05 |
| Sex | .00 |
| Educational level | .13 |
| ***Adolescent legitimacy beliefs*** | .16 |
| ***Adolescent personality traits*** |  |
| Environmental sensitivity | -.14 |
| Neuroticism | -.05 |

*Note*. Cramer’s V is reported for group differences in sex and educational levels (chi-square test). All others are cohen’s *d* (*t*-test). All effect sizes were not significant (*p* > .05).

**Tabel S2**

*Correlations with Parenting-Affect Density*

|  | *r / d / η²* |
| --- | --- |
| ***Average levels of parenting*** |  |
| Warmth | -.15 |
| Autonomy support | -.08 |
| Psychological control | -.14 |
| Strictness | -.07 |
| Monitoring | -.07 |
| ***Average levels of affect*** |  |
| Positive affect | -.01 |
| Negative affect | -.11 |
| ***Adolescent psychological functioning*** |  |
| Depressive symptoms | -.01 |
| Anxiety symptoms | .08 |
| Self-esteem | .08 |
| ***Adolescent demographics*** |  |
| Age | -.04 |
| Sex | -.23 |
| Educational level | .03 |
| ***Adolescent legitimacy beliefs*** | .12 |
| ***Adolescent personality traits*** |  |
| Environmental sensitivity | -.03 |
| Neuroticism | -.02 |

*Note*. Cohen’s *d* is reported for sex (1 = male, 2 = female). Eta squared is reported for education (1 = low, 2 = moderate, 3 = high). All other are correlations. All effect sizes were not significant (*p* > .05).

**Supplementary Information 2
Network Plots of the Five Families Who Were Placed in a Subgroup by Themselves**

*
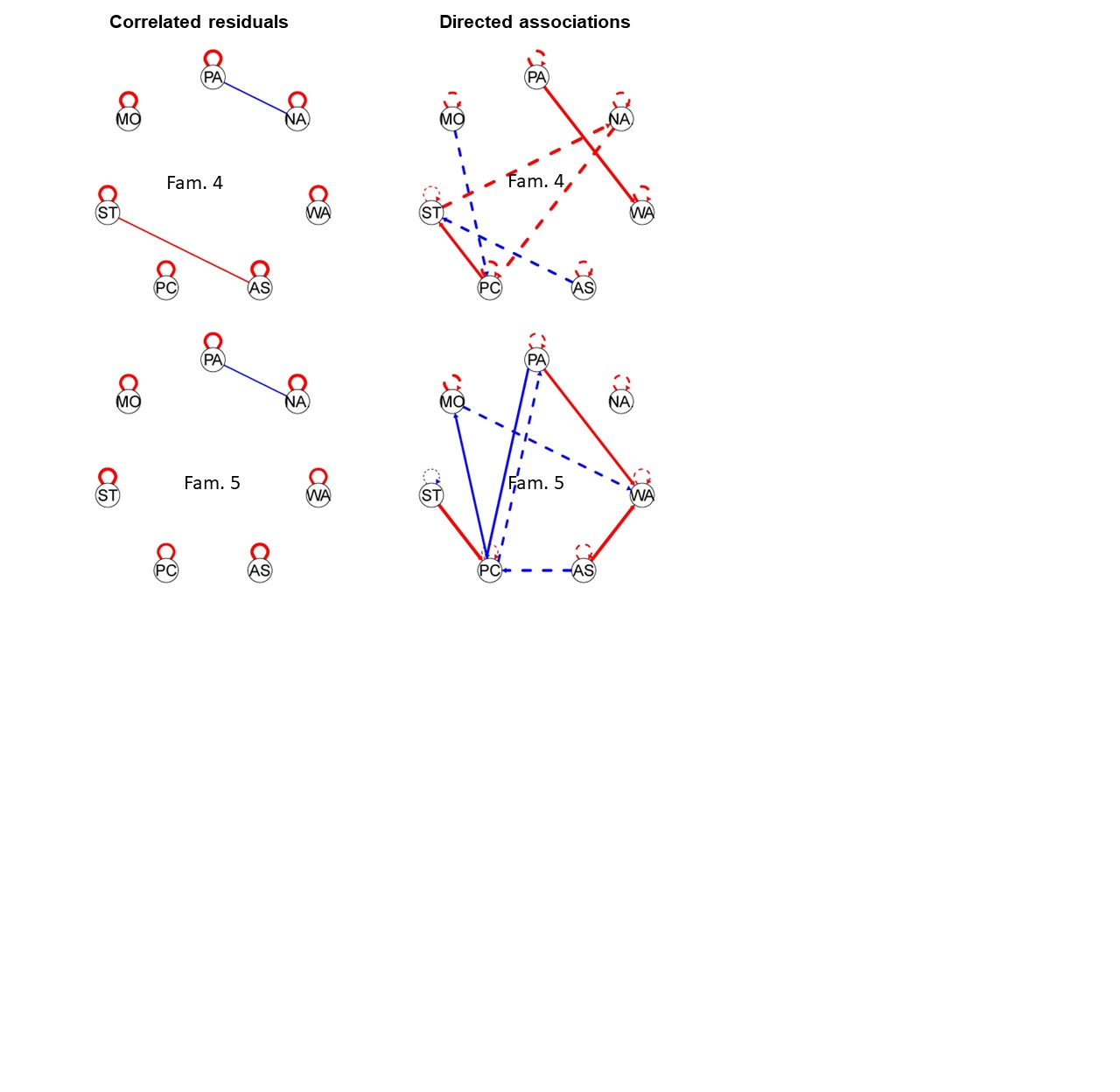
***Figure S1***Family-specific networks of the five families who were placed in a subgroup by themselves***
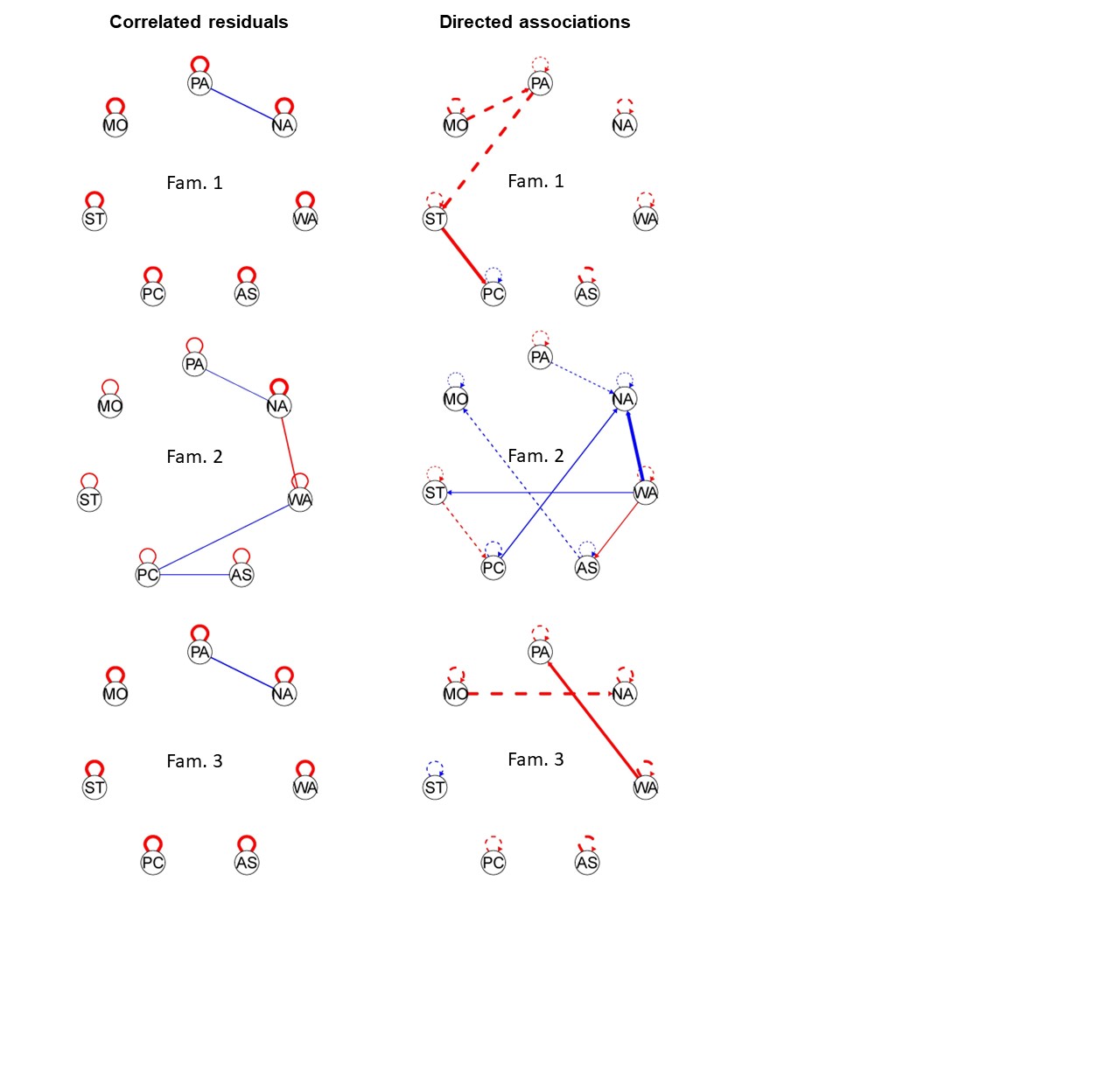
**

*Note*. Hybrid-GIMME allowed to model undirected contemporaneous associations (i.e., correlated residuals) and directed contemporaneous or lagged associations. The solid lines reflect contemporaneous (same-day) associations, dashed lines lagged (next-day) associations, red lines positive associations, and blue lines negative associations. Model fit: Family 1 (χ^2^(59) = 62.30, *p* = .360, RMSEA = .02, SRMR = .07, NNFI = .93, CFI = .96), Family 2 (χ^2^(52) = 568.60, *p* = .299, RMSEA = .03, SRMR = .08, NNFI = .93, CFI = .96), Family 3 (χ^2^(60) = 548.26, *p* = .665, RMSEA = .00, SRMR = .07, NNFI = 1.0, CFI = 1.0). Family 4 (χ^2^(55) = 563.50, *p* = .424, RMSEA = .01, SRMR = .07, NNFI = .98, CFI = .99), Family 5 (χ^2^(54) = 566.36, *p* = .377, RMSEA = .02, SRMR = .07, NNFI = .95, CFI = .97). PA = positive affect. NA = negative affect. WA = warmth. AS = autonomy support. PC = psychological control. ST = strictness. MO = monitoring.

**Supplementary Information 3
Plots of the Sensitivity Analyses**

**Figure S2**

*Summary plot of the sample (n = 124)*

| **Correlated residuals** | **Directed associations** |
| --- | --- |
| **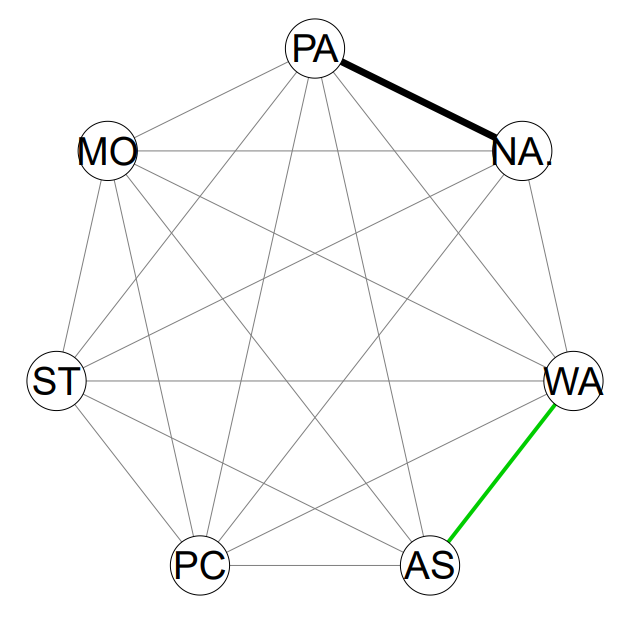** | **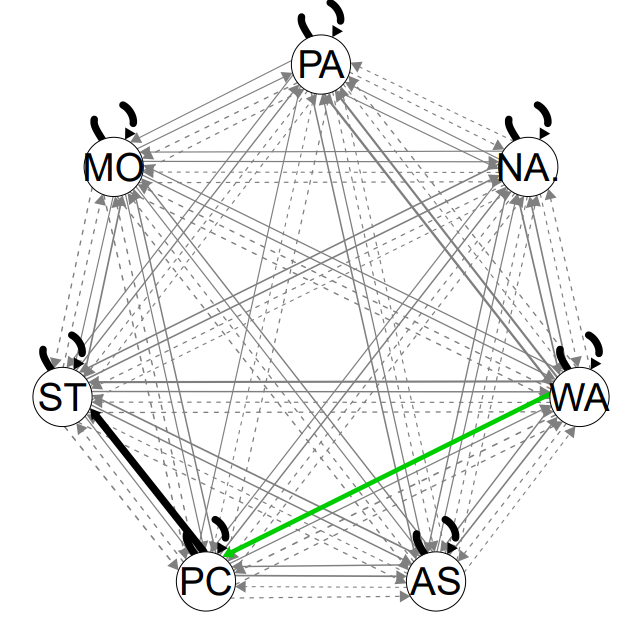** |

*Note*. Hybrid-GIMME allowed to model undirected same-day associations (on the left) and directed same- or next-day associations (on the right). On the left: Black line between adolescent positive and negative affect is a same-day association estimated for everyone in the sample. On the right: Black line from parental psychological control to parental strictness is a same-day association estimated for everyone in the sample. Several subgroup-specific associations were found, which are depicted green lines. The grey lines in both figures are individual-level associations found for one or some individual families in the sample, with line thickness corresponding to the number of families for which that association was estimated. The arrows indicate the directionality of the association. PA = positive affect. NA = negative affect. WA = warmth. AS = autonomy support. PC = psychological control. ST = strictness. MO = monitoring.

**Figure S3**

*Summary plot of subgroup 1 (n = 64)*

| **Correlated residuals** | **Directed associations** |
| --- | --- |
| **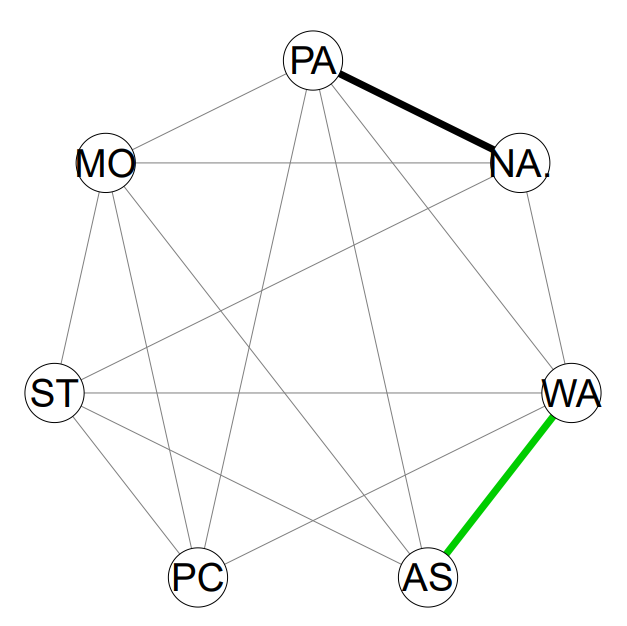** | **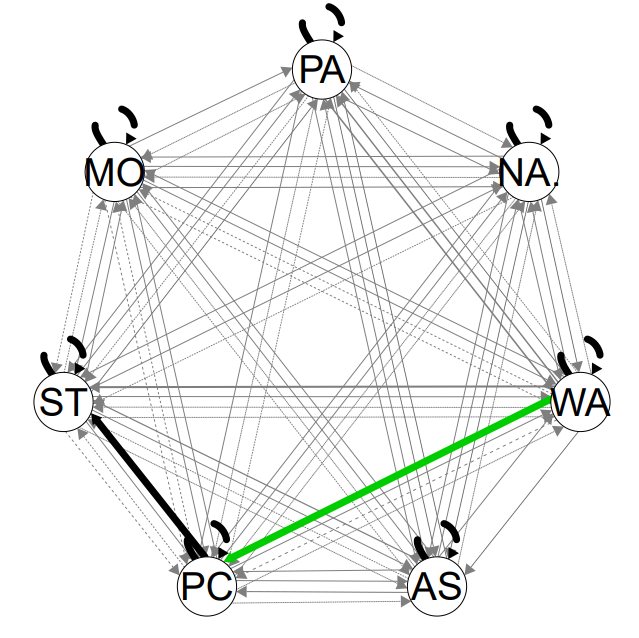** |

*Note*. Hybrid-GIMME allowed to model undirected same-day associations (left figure) and directed same- or next-day associations (right figure). On the left: Black line between adolescent positive and negative affect is a same-day association estimated for everyone in the sample and thus also in this subgroup. On the right: Black line from parental psychological control to parental strictness is a same-day association estimated for everyone in the sample and thus also in this subgroup. Both figures: The green lines are subgroup-specific associations estimated for everyone in this subgroup. The grey lines are individual-level associations found for one or some individual families in this subgroup, with line thickness corresponding to the number of families for which that association was estimated. The arrows indicate the directionality of the association. PA = positive affect. NA = negative affect. WA = warmth. AS = autonomy support. PC = psychological control. ST = strictness. MO = monitoring.

**Figure S4**

*Summary plot of subgroup 2 (n = 57***)**

| **Correlated residuals** | **Directed associations** |
| --- | --- |
| **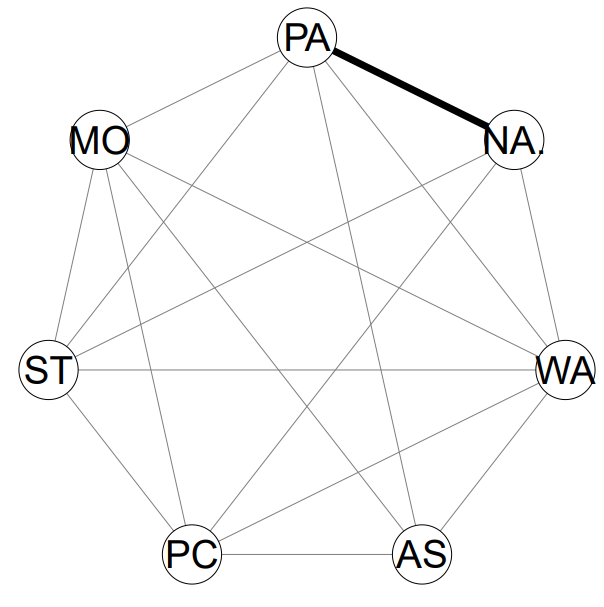** | **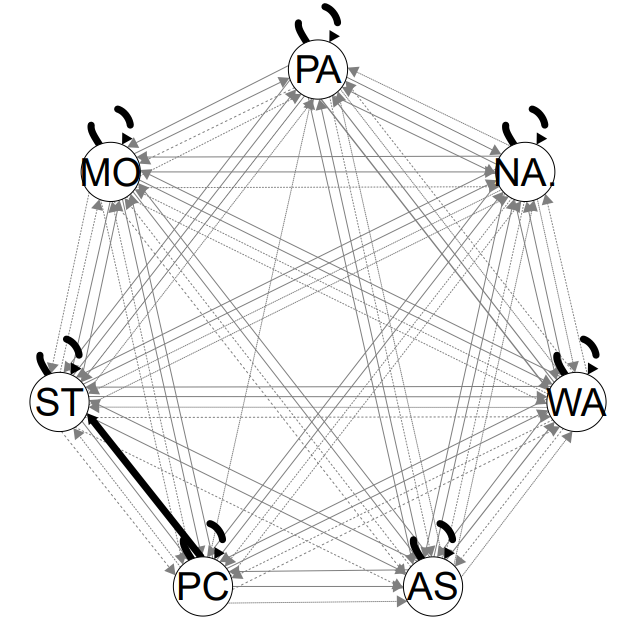** |

*Note*. Hybrid-GIMME allowed to model undirected same-day associations (left figure) and directed same- or next-day associations (right figure). On the left: Black line between adolescent positive and negative affect is a same-day association estimated for everyone in the sample and thus also in this subgroup. On the right: Black line from parental psychological control to parental strictness is a same-day association estimated for everyone in the sample and thus also in this subgroup. No subgroup-specific associations were found and therefore no green lines were depicted in either figure. The grey lines in both figures are individual-level associations found for one or some individual families in this subgroup, with line thickness corresponding to the number of families for which that association was estimated. The arrows indicate the directionality of the association. PA = positive affect. NA = negative affect. WA = warmth. AS = autonomy support. PC = psychological control. ST = strictness. MO = monitoring.
